# Intracranial Electrophysiology of Auditory Selective Attention Associated with Speech Classification Tasks

**DOI:** 10.3389/fnhum.2016.00691

**Published:** 2017-01-10

**Authors:** Kirill V. Nourski, Mitchell Steinschneider, Ariane E. Rhone, Matthew A. Howard III

**Affiliations:** ^1^Human Brain Research Laboratory, Department of Neurosurgery, The University of IowaIowa City, IA, USA; ^2^Departments of Neurology and Neuroscience, Albert Einstein College of MedicineBronx, NY, USA; ^3^Pappajohn Biomedical Institute, The University of IowaIowa City, IA, USA

**Keywords:** auditory cortex, electrocorticography, Heschl’s gyrus, high gamma, middle temporal gyrus, prefrontal cortex, speech, supramarginal gyrus

## Abstract

Auditory selective attention paradigms are powerful tools for elucidating the various stages of speech processing. This study examined electrocorticographic activation during target detection tasks within and beyond auditory cortex. Subjects were nine neurosurgical patients undergoing chronic invasive monitoring for treatment of medically refractory epilepsy. Four subjects had left hemisphere electrode coverage, four had right coverage and one had bilateral coverage. Stimuli were 300 ms complex tones or monosyllabic words, each spoken by a different male or female talker. Subjects were instructed to press a button whenever they heard a target corresponding to a specific stimulus category (e.g., tones, animals, numbers). High gamma (70–150 Hz) activity was simultaneously recorded from Heschl’s gyrus (HG), superior, middle temporal and supramarginal gyri (STG, MTG, SMG), as well as prefrontal cortex (PFC). Data analysis focused on: (1) task effects (non-target words in tone detection vs. semantic categorization task); and (2) target effects (words as target vs. non-target during semantic classification). Responses within posteromedial HG (auditory core cortex) were minimally modulated by task and target. Non-core auditory cortex (anterolateral HG and lateral STG) exhibited sensitivity to task, with a smaller proportion of sites showing target effects. Auditory-related areas (MTG and SMG) and PFC showed both target and, to a lesser extent, task effects, that occurred later than those in the auditory cortex. Significant task and target effects were more prominent in the left hemisphere than in the right. Findings demonstrate a hierarchical organization of speech processing during auditory selective attention.

## Introduction

Auditory selective attention is a crucial process for extracting ecologically relevant information from complex acoustic environments. It has been defined as the process by which specific features in the acoustic environment are emphasized at the expense of other features that are not specifically related to the perceptual task at hand (Egeth, 1967). Auditory selective attention is dysfunctional in multiple clinical populations including attention deficit hyperactivity disorder, major depression, schizophrenia and specific language impairment (e.g., Noterdaeme et al., 2001; Scholes and Martin-Iverson, 2010; Gomes et al., 2012; Greimel et al., 2015). This clinical relevance requires better understanding of the neural underpinnings of auditory selective attention in order to optimize therapeutic interventions.

Two basic paradigms have been generally utilized to study auditory selective attention. The most common approach relies on attending to one stream of sounds embedded in a complex multi-stream environment. Classically, this paradigm has been referred to as the cocktail party effect, exemplified by focusing attention to a given talker within a multi-talker environment (Cherry, 1953; Bregman, 1990). An extensive literature has been devoted to understanding the neural mechanisms underlying this effect. Non-invasive studies examining auditory averaged evoked potentials (AEPs) and neuromagnetic fields have demonstrated enhanced responses to components of the attended stream and dampened responses to the simultaneously presented unattended stream (e.g., Ding and Simon, 2012; Simon, 2015). Functional neuroimaging studies have similarly shown that activity within regions of the auditory cortex involved in processing a specific acoustic stream is enhanced when attention is directed to that stream (Giard et al., 2000; Paltoglou et al., 2009, 2011; Lee et al., 2013; Alho et al., 2014).

The second paradigm examining auditory selective attention uses a single stream of sound wherein focus is directed towards specific features of the stream at the expense of other features within the same stream. Variants of this paradigm have been extensively used to study mechanisms of visual attention (for review, see Lee et al., 2014), and its use offers several advantages when examining auditory speech processing (Hugdahl et al., 2003). These advantages include a more controlled examination of phonemic and semantic tiers of speech processing unencumbered by the additional task demands required when speech is presented in a complex multi-stream environment (e.g., Paltoglou et al., 2011). Many auditory studies using this paradigm demonstrated that activity within specific regions of auditory cortex was enhanced when a specific stimulus attribute was a target vs. when the same stimulus was presented during passive listening (e.g., Hugdahl et al., 2003; Paltoglou et al., 2009; Woods et al., 2010, 2011; Alho et al., 2014).

Despite the important findings obtained using non-invasive methods, limitations inherent to indirect measures of neural activity hamper detailed understanding of the mechanisms underlying auditory selective attention within the human brain (Giard et al., 2000; Lee et al., 2014). AEPs and neuromagnetic fields are limited in their ability to identify the specific brain regions modulated by auditory selective attention. Functional magnetic resonance imaging (fMRI) and other neuroimaging techniques provide an indirect measure of neural activity and poorly reflect the timing of cortical activation. Additionally, scanner noise during fMRI studies of sound processing remains a significant issue (Lee et al., 2014). Recordings directly from the human brain overcome these limitations and are possible in neurosurgical patients who undergo implantation of electrodes for clinical diagnostic purposes (e.g., Nourski and Howard, 2015). The strength of these direct recordings for examining auditory selective attention is exemplified by the study of Mesgarani and Chang (2012), which unequivocally demonstrated enhancement of neural activity related to the attended stream at the expense of the ignored stream within a multitalker environment. The robust nature of auditory activity modulated by selective attention was further emphasized in a study of spatial selective attention, where activity recorded from a single electrode over the superior temporal gyrus (STG) was sufficient to predict the attended stream with above-chance accuracy (Dijkstra et al., 2015).

Intracranial studies have also examined auditory selective attention using single-stream target detection tasks (Chang et al., 2011; Steinschneider et al., 2014). In the case of the former study, modest enhancement of high gamma (>70 Hz) activity was observed on the lateral surface of the STG in response to syllables that were the target. In the case of the latter study, responses to monosyllabic words recorded from the STG were modulated by task condition, but not by behavioral performance. In contrast, responses recorded from prefrontal cortex (PFC) also were related to task condition, but did co-vary with behavior. It was concluded that the STG represents a relatively early stage in the neural encoding of words, whereas activity in PFC is a later processing stage required to make behavioral decisions based on the task condition.

While both studies utilizing direct intracranial recordings highlight the strengths of this approach in identifying speech processing mechanisms while subjects performed auditory selective attention tasks, they had several limitations. In the study of Chang et al. (2011), only phonemic target tasks were used. However, the ultimate goal of speech processing is higher-order decoding of lexical-semantic content. The study of Steinschneider et al. (2014), which did examine semantic processing, did not examine activity within auditory-related cortex surrounding STG including middle temporal and supramarginal gyri (MTG, SMG). These regions have been shown to be involved in decoding the semantic content of words (e.g., Corina et al., 2010). Further, the limited number of subjects precluded examination of hemispheric differences that might occur during semantic classification tasks. Current models of speech and language processing posit bilateral activation of the STG, but it is unclear whether a hemispheric asymmetry in activation at this level would emerge when subjects perform a semantic classification task (Hickok, 2009; Cogan et al., 2014). These same models posit emergence of hemispheric differences at higher levels of processing requiring semantic classification. Understanding hemispheric specialization for this level of processing with intracranial recordings has been hampered by either examination of only the language-dominant hemisphere (e.g., Chang et al., 2011) or small subject cohorts that do not provide sufficient statistical power for hemispheric comparisons (e.g., Steinschneider et al., 2014).

In the present study, we sought to address these issues by utilizing target detection tasks that require acoustic, phonemic and semantic processing, compared to tasks where the latter two levels of processing were not required (i.e., tone targets). Task-related effects were defined in the present study as differential representation of non-target words during tone-target vs. semantic- target tasks. Likewise, target effects were defined as differential representation of target and non-target words in semantic categorization tasks. We extended analyses to include core auditory cortex in posteromedial Heschl’s gyrus (HG), non-core auditory cortex (anterolateral HG and STG), auditory-related areas (MTG, SMG) and PFC (inferior and middle frontal gyri; IFG and MFG, respectively) in a larger subject cohort that included both language-dominant and non-dominant hemispheres.

When examining human intracranial data, it is possible to focus analysis on multiple frequency bands within the electrocorticogram. As an initial step to this multiband analysis, this study focuses on high gamma activity (70–150 Hz). Multiple studies have demonstrated the relevance of this frequency band for examining neural mechanisms of auditory cortical processing (e.g., Crone et al., 2001, 2006; Brugge et al., 2009; Edwards et al., 2009; Mesgarani and Chang, 2012; Steinschneider et al., 2014; Nourski and Howard, 2015). High gamma activity has been directly related to acoustic-phonemic transformations at the level of the STG, which would be a key process required for tasks used in the present study (Mesgarani et al., 2014; Moses et al., 2016). Further, functional neuroimaging studies have demonstrated a positive correlation between high gamma activity and hemodynamic responses (Nir et al., 2007; Whittingstall and Logothetis, 2009). Invasive electrophysiological studies in animals have established that high gamma is the frequency band most closely related to unit activity (Ray et al., 2008; Steinschneider et al., 2008). Finally, the present study builds upon high gamma analysis in our previous work examining target detection tasks (Steinschneider et al., 2014; Nourski et al., 2015).

## Materials and Methods

### Subjects

Experimental subjects were nine neurosurgical patients (3 female, 6 male, age 21–51 years old, median age 33 years old) diagnosed with medically refractory epilepsy. Two subjects (L292 and R334) were left-handed, while all other subjects were right handed. All subjects were left hemisphere language-dominant except L292, who had right language dominance, as determined by intracarotid amytal (Wada) test. The patients were undergoing chronic invasive electrocorticographic monitoring to identify potentially resectable seizure foci. Research protocols were approved by the University of Iowa Institutional Review Board and the National Institutes of Health. Written informed consent was obtained from all subjects. Research participation did not interfere with acquisition of clinically required data, and subjects could rescind consent at any time without interrupting their clinical evaluation.

All subjects underwent audiometric evaluation before the study, and none was found to have hearing deficits that should impact the findings presented in this study. All subjects except three (L258, L307 and R334) had pure-tone thresholds within 25 dB hearing level (HL) between 250 Hz and 4 kHz. Subject L258 had a mild low-frequency hearing loss (at 250 Hz, thresholds were 30 and 25 HL for left and right ear, respectively). Subjects L307 and R334 had notches at 4 kHz (40 and 35 dB HL, respectively, both in right ear only). Word recognition scores, as evaluated by spondees presented via monitored live voice, were 88% and 96% in subject R334 (right and left ear, respectively), and ≥96% in all other tested subjects. Speech reception thresholds were within 15 dB HL in all tested subjects, including those with tone audiometry thresholds outside the 25 dB HL criterion.

All subjects were native English speakers, with the exception of subject L275, who was a native Bosnian speaker who learned German at the age of 10 and English at the age of 17. Subject L258 had mild deficiencies in verbal working memory, as revealed during formal neuropsychological testing. Subject L275 had grossly intact conversational language comprehension, though neuropsychological testing showed non-localizing cognitive function deficits. Subject R334 had impaired visual memory and low processing speed (in contrast to strong verbal memory). All subjects adequately performed the experimental tasks both in terms of target detection accuracy and reaction times (RTs). Intracranial recordings revealed that cortical sites within HG, STG, MTG, SMG, IFG and MFG examined in the present study were not epileptic foci in any subject.

### Procedure

Experiments were carried out in a dedicated electrically-shielded suite in The University of Iowa Clinical Research Unit. The room was quiet, with lights dimmed. Subjects were awake and reclining in a hospital bed or an armchair. Experimental stimuli (tones and speech syllables; see below) were presented in random order in multiple target detection tasks. The target stimuli included complex tones (presented as first block in each subject), and words belonging to a specific semantic category (animals and numbers). Each recording block was associated with a single target detection task and included 200 or 210 trials. The target-to-nontarget trial ratio was 40/160 in the tone detection block and 45/165 in semantic categorization blocks (due to addition of 10 novel words in these blocks, as described below). The simpler tone detection task was always presented prior to the more demanding semantic tasks to disambiguate order effects from those associated with semantic processing (see Hugdahl et al., 2003; Nourski et al., 2015). Prior to data collection, the subjects were presented with a random-sequence preview of stimuli to ensure that the sounds were presented at a comfortable level and that they understood the task requirements.

Subjects were instructed to use the index finger ipsilateral to the hemisphere from which recordings were made to report their behavioral responses in target detection tasks (subject B335, who had bilateral electrode coverage, used his right index finger). This was done to minimize contributions of neural activity reflecting preparatory, motor and somatosensory responses associated with the button press as opposed to auditory, speech and language processing. A similar rationale for separating cortical activity related to the attentional task vs. motor activity related to the behavioral response has been made for non-invasive neuroimaging studies (Lee et al., 2014).

### Stimuli

The present study utilized the experimental paradigm used previously by Steinschneider et al. (2014). Standard experimental stimuli were monosyllabic consonant-vowel-consonant words “cat”, “dog”, “five”, “ten”, “red” and “white”, from TIMIT (Garofolo et al., 1993) and LibriVox^1^ databases. Twenty unique exemplars of each syllable were used in each experiment: 14 were spoken by different male and six were spoken by different female talkers. Additionally, the stimulus set included two complex tones with fundamental frequencies of 125 and 250 Hz, approximating the average voice fundamental frequencies of male and female speakers, respectively. The 125 and 250 Hz complex tones were presented in each task for a total of 28 and 12 trials, respectively, matching the ratio between words spoken by male and female talkers.

In two subjects (L258 and L275), two additional sets of non-word syllables were added to the stimulus corpus described above and were used in all experiments in these two subjects. The stimuli were syllables “res”, and “tem” (20 exemplars of each; 14 by male, 6 by female talkers). The non-word syllables were excised from real English words present in TIMIT or LibriVox recordings using SoundForge 4.5 (Sonic Foundry Inc., Madison, WI, USA). This was done in part to reduce the subjects’ reliance on early acoustic/phonemic cues to identify targets. For example, inclusion of “tem” precluded the possibility for the subjects to rely solely on the initial /t/ in the detection of “ten” in the number target detection task. Instead, the subjects had to make their decision based on processing of the entire syllable including the final consonant.

In all other subjects, each of the two semantic categorization blocks included ten additional exemplars of novel words (five targets and five non-targets), all spoken by male talkers. In the animal target block, the five novel target stimuli were “bat”, “duck”, “goat”, “pig” and “rat”, balanced by five additional non-target novel stimuli “bite”, “give”, “read”, “run” and “walk”. In the number target block, the five novel target stimuli were “one”, “three”, “four”, “six” and “nine”, balanced by five additional non-target novel stimuli “hit”, “make”, “kiss”, “sit” and “wait”. This was done in part to minimize the subjects’ possible reliance solely on acoustic and phonemic cues to identify targets. Additionally, the non-target novel stimuli were action words and were included in part to pilot studies examining whether action words are processed differentially from nouns in spatially distinct cortical regions. Yet another reason for the inclusion of these stimuli was to test whether novel stimuli *per se* were processed differentially from standard, frequent, stimuli, possibly to identify the neural substrates of the automatic endogenous P3a component generated in the frontal regions of the brain. These comparisons were beyond the scope of the present study, and will be reported in a subsequent research article.

All stimuli were normalized to the same root-mean-square amplitude and edited to be 300 ms in duration using SoundForge with 5 ms rise-fall times. They were presented with an inter-stimulus interval chosen randomly within a Gaussian distribution (mean interval 2 s; SD = 10 ms) to reduce heterodyning in the recordings secondary to power line noise. Stimuli were delivered via insert earphones (ER4B, Etymotic Research, Elk Grove Village, IL, USA) that were integrated into custom-fit earmolds. Stimulus delivery was controlled using Presentation software (Version 16.5 Neurobehavioral Systems^2^).

### Recording

ECoG recordings were simultaneously made from HG and lateral hemispheric surface using multicontact depth and subdural grid electrodes, respectively (Ad-Tech Medical, Racine, WI, USA). Details of electrode implantation have been described previously, and more comprehensive details regarding recording, extraction and analysis of high gamma cortical activity are available for the interested reader (Howard et al., 1996, 2000; Reddy et al., 2010; Nourski et al., 2013; Nourski and Howard, 2015). In brief, hybrid depth electrode arrays were implanted stereotactically into HG, along its anterolateral to posteromedial axis. In subject L258, a hybrid depth electrode was used, which contained four cylindrical platinum macro-contacts, spaced 10 mm apart, and 14 platinum micro-contacts, distributed at 2–4 mm intervals between the macro contacts. In all other subjects, depth electrodes with eight macro-contacts, spaced 5 mm apart, were used. In subjects L282 and R334, two depth electrodes were implanted in the left and right superior temporal plane, respectively, providing additional HG coverage. In subject B335, depth electrodes were implanted in both the left and the right hemisphere. Subdural grid arrays were implanted over the lateral surface of temporal and frontal lobes. The grid arrays consisted of platinum-iridium disc electrodes (2.3 mm exposed diameter) embedded in a silicon membrane and arranged in a 2 × 8, 4 × 8 or 8 × 12 configuration with 5 or 10 mm center-to-center inter-electrode distance. A subgaleal contact was used as a reference. Electrode arrays were placed solely on the basis of clinical requirements, and were part of a more extensive set of recording arrays meant to identify seizure foci. Electrodes remained in place under the direction of the patients’ treating neurologists.

Reconstruction of the anatomical locations of the implanted electrodes and their mapping onto a standardized set of coordinates across subjects was performed using FreeSurfer image analysis suite and in-house software, as described in detail in Nourski et al. (2014). In brief, subjects underwent whole-brain high-resolution T1-weighted structural MRI scans (resolution 0.78 mm × 0.78 mm, slice thickness 1.0 mm) before electrode implantation. Two volumes were averaged to improve the signal-to-noise ratio of the MRI data sets and minimize the effects of movement artifact on image quality. After electrode implantation, subjects underwent MRI and thin-slice volumetric computed tomography (resolution 0.51 mm × 0.51 mm, slice thickness 1.0 mm) scans. Contact locations of the HG depth electrodes and subdural grid electrodes were first extracted from post-implantation MRI and computed tomography scans, respectively. These were then projected onto preoperative MRI scans using non-linear three-dimensional thin-plate spline morphing, aided by intraoperative photographs. Finally, these were then projected into the standard Montreal Neurological Institute (MNI) space (MNI305) using surface-based warping.

Recording sites were included in analyses based on their anatomical location (i.e., implanted in the gray matter of the HG or overlying the lateral surface of the STG, MTG, SMG, IFG or MFG). Anatomical location was determined by the localization of each electrode in the pre-implantation MRI for each subject individually, and not based on the common MNI coordinates. In each subject, HG was subdivided into posteromedial and anterolateral portions based on physiological criteria (Brugge et al., 2008, 2009). Specifically, recording sites were assigned to posteromedial HG if they exhibited phase-locked ECoG responses to 100 Hz click trains and AEPs to these stimuli featured short-latency (<20 ms) components. Such response features are not present within anterolateral HG.

Data acquisition was controlled by a TDT RZ2 real-time processor (Tucker-Davis Technologies, Alachua, FL, USA). Collected ECoG data were amplified, filtered (0.7–800 Hz bandpass, 12 dB/octave rolloff), digitized at a sampling rate of 2034.5 Hz and stored for subsequent offline analysis. Behavioral responses to the target stimuli were recorded using a Microsoft SideWinder game controller. The timing of the button-press events was recorded and stored for analysis along with ECoG data.

### Analysis

RTs (i.e., timing of the button presses relative to the onset of the target stimulus) were measured using a 1.8 s maximum RT criterion, which corresponded to the 98.6th percentile of the overall RT sample. This led to the rejection of two trials in L258, one trial in L275, two trials in R288 and four trials in R334, which were considered outliers in terms of behavioral performance.

ECoG data obtained from each recording site were downsampled to a rate of 1000 Hz. To minimize contamination with power line noise, ECoG waveforms were de-noised using an adaptive notch filtering procedure (Nourski et al., 2013). Individual trials were screened for possible contamination from electrical interference, epileptiform spikes, high amplitude slow wave activity, or movement artifacts. To that end, individual trial waveforms with voltage exceeding five standard deviations from the mean were rejected from further analysis. Data analysis was performed using custom software written in MATLAB Version 8.3 programming environment (MathWorks, Natick, MA, USA).

Quantitative analysis of ECoG focused on the high gamma frequency band (70–150 Hz) and was implemented using a demodulated band transform-based algorithm (Kovach and Gander, 2016). This was done by computing the discrete Fourier transform over the entire duration of each recording block and segmenting the discrete Fourier transform over positive frequencies into windows of 20 Hz bandwidth. Event-related band power (ERBP) was calculated by log-transforming power for each center frequency and normalizing it to a baseline value measured as the mean power in the prestimulus reference interval (100–200 ms before stimulus onset). The resultant high gamma power waveforms were averaged across trials and, for plotting purposes, smoothed using a moving average filter with a span of 25 ms.

The presence of task- and target-related modulation of high gamma activity was assessed for each cortical site. First, across-trial mean ERBP and its 95% confidence interval were measured for all recording sites within the six regions of interest (posteromedial HG, anterolateral HG, STG, MTG, SMG, PFC) and for each of the three experimental conditions (non-target word in tone or semantic task, or target). Missed target trials were excluded from the target condition. Responses were considered significant if the lower limit of high gamma ERBP 95% confidence interval exceeded 0 dB relative to the prestimulus mean within 750 ms after stimulus onset and remained positive for at least 50 ms (see Nourski et al., 2014). To decrease the number of multiple comparisons, sites that did not exhibit a significant high gamma response to words presented in any of the three experimental conditions, were excluded from further analyses of task and target effects.

For sites that exhibited significant responses, high gamma ERBP was averaged in 50 ms-wide consecutive bins from 50–100 ms to 500–550 ms (in subjects L258, L307, R316 and B335), 550–600 ms (in subjects R288 and R320), 600–650 ms (in subjects L292), 650–700 ms (in subject L275), or 700–750 ms (in subject R334). The number of analysis bins varied across subjects to avoid overlapping with activity occurring after the behavioral responses as determined by RTs to target stimuli.

Two-sample one-tailed *t*-tests were performed on single-trial binned ERBP values to compare responses to non-target animal and number words presented in the tones vs. semantic task (task effect), and non-target vs. target animal and number words presented in the semantic task (target effect). Multiple comparison correction was done using false discovery rate (FDR) approach (Benjamini and Hochberg, 1995; Storey, 2002) as implemented in MATLAB Version 8.3. Task and target effects were quantified at a *p* = 0.05 significance level. A significant difference in high gamma ERBP between the two non-target conditions or between semantic non-target and target condition within at least one 50 ms bin was interpreted as a task or target effect, respectively. The timing of task- and target-related effects was quantified by computing the number of sites within each region of interest that exhibited either effect in each of the 50 ms bins.

## Results

### Behavioral Performance

**Figure 1:**
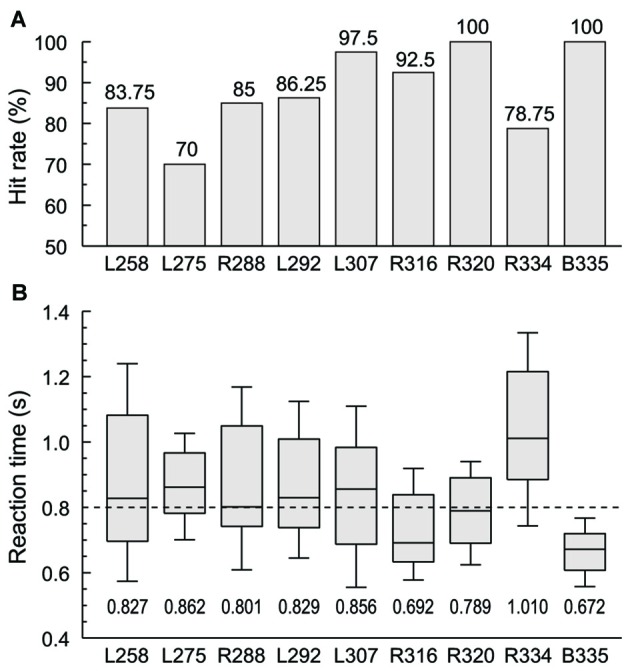
**Subjects’ behavioral performance in the semantic categorization tasks. (A)** Hit rates (% correctly detected target stimuli) for each of the nine subjects. **(B)** Reaction times (RTs). Box-and-whiskers plots depict median values and 10th, 25th, 75th and 90th percentiles; median values are shown for each subject underneath the plots. Dashed line corresponds to the grand median value (800 ms). Behavioral data from the two semantic categorization tasks (animals and numbers target category) were combined in each subject.

Subjects were required to respond with a button press when they were presented with words in the category of animals (“cat” and “dog”) in one experimental block or numbers (“five” and “ten”) in the other. Behavioral performance in these two semantic tasks ranged from 70% to 100% hit rate across the nine subjects, and was not significantly different across the four target words (Figure 1A). RTs varied widely across subjects, with a median RT of 0.8 s (Figure 1B). The grand median was within the interquartile range of RTs in all subjects except two. Subject R334 tended to respond slower both to this task and in general conversation, while B335 had faster responses compared to the rest of the subjects. There was no significant difference in RT between the two blocks in any of the subjects with the exception of L258, whose performance was significantly slower in the numbers block compared to the animals block (median RTs 0.984 and 0.714 s, respectively, *p* < 0.001, Wilcoxon rank sum test, FDR-corrected).

### HG

**Figure 2:**
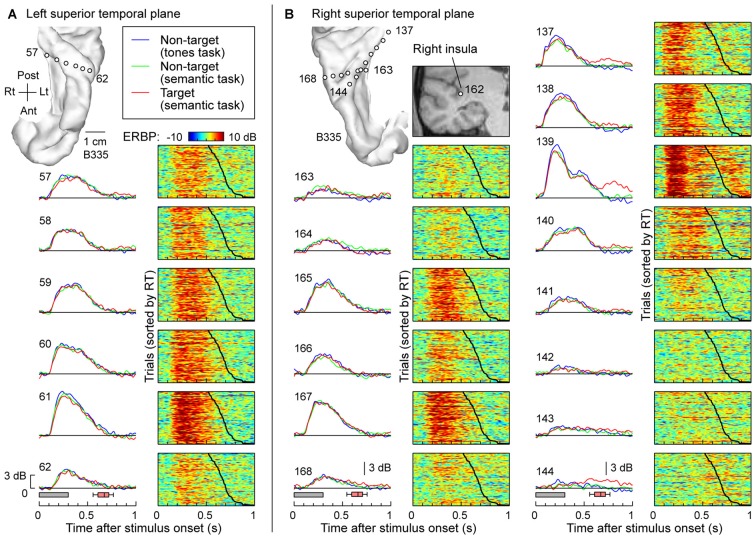
**High gamma responses to words “cat”, “dog”, “five” and “ten” recorded from the Heschl’s gyrus (HG) in subject B335. (A)** Responses recorded from a depth electrode that targeted the left HG (contacts 57–62). Magnetic resonance imaging (MRI) reconstruction of the top-down view of left superior temporal plane depicting the location of the recording sites is shown in the top left corner. Left column: high gamma event-related band power (ERBP) waveforms, averaged across trials where the stimuli were non-targets in a tone detection task, non-targets in a semantic categorization task and targets in a semantic categorization tasks (plotted in blue, green and red, respectively). Gray boxes underneath high gamma waveforms represent stimulus duration (300 ms); pink box-and-whiskers plots denote the timing of button presses to the target stimuli (median, 10th, 25th, 75th and 90th percentile; same convention used for subsequent figures). Right column: waterfall plots showing single-trial high gamma ERBP in response to the target stimuli. Trials are sorted by RT (top to bottom: shortest to longest). Thick black line indicates RTs for each trial. **(B)** Responses recorded from two depth electrodes that targeted the right insula (contact 162) traversing planum temporale (contacts 163–168) and right HG (contacts 137–144). MRI reconstruction of the top-down view of right superior temporal plane showing the location of the recording sites is shown on the top left. Inset: MRI coronal section through the right temporal cortex showing location of contact 162 that targeted the insula. Average high gamma ERBP waveforms and single-trial high gamma waterfall plots are depicted for each site in the superior temporal plane as in **(A)**. Note that a click was associated with the button press and was likely responsible for target-specific activity seen on contacts within core auditory cortex (e.g., contact 139).

The typical pattern observed within posteromedial regions of HG was characterized by robust responses to the speech stimuli regardless of task and whether the words were targets or not. This is exemplified in Figure 2, which depicts responses recorded simultaneously from multiple sites implanted in and around HG of both hemispheres in one subject (B335). Figure 2A depicts responses from the six recording sites in HG of the left (language-dominant) hemisphere. High gamma ERBP envelopes averaged across all trials are shown in the left column. Stimulus duration and the timing of behavioral responses to target stimuli (button presses) are indicated below by gray rectangles and red box-and-whiskers plots, respectively. Comparable high gamma responses to the words were elicited irrespective of task (tone detection vs. semantic tasks) or target status (semantic tasks). The waterfall plots in the right-hand column of Figure 2A show high gamma ERBP on a trial-by-trial basis, sorted according to the RT (indicated by the black line; fastest responses on the top). Increases in high gamma ERBP dissipated prior to the subject’s behavioral response and did not systematically vary with RT. As previously reported, sites overlying the most anterolateral portion of HG were generally characterized by weaker responses with longer onset latencies when compared to those in more posteromedial regions of the gyrus (i.e., putative auditory core cortex; e.g., Brugge et al., 2008; Nourski et al., 2014).

**Figure 3:**
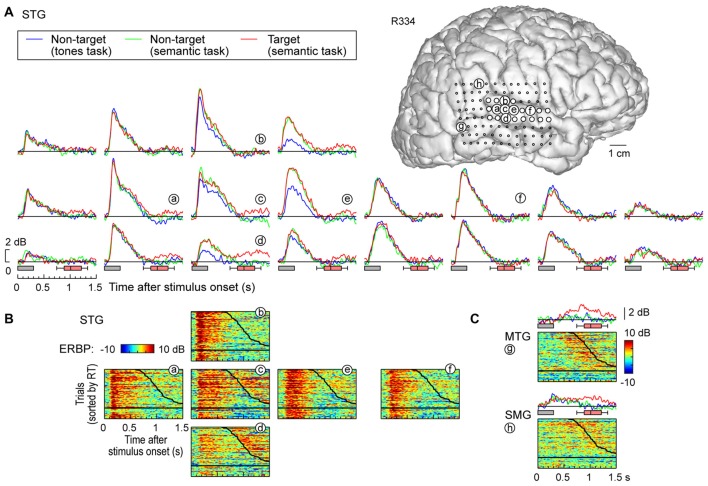
**High gamma responses recorded from right lateral temporal cortex in subject R334. (A)** Average high gamma ERBP waveforms for superior temporal gyrus (STG) sites, plotted for trials when words (“cat”, “dog”, “five” and “ten”) were non-targets in a tone detection task, non-targets in a semantic categorization task and targets in a semantic categorization tasks (blue, green and red waveforms, respectively). MRI reconstruction of the lateral view of the right cerebral hemisphere depicting the location of the recording sites is shown at the top right. **(B)** Waterfall plots showing single-trial high gamma ERBP in response to the target stimuli for six representative sites on the STG **(a–f)**. Trials are sorted by RT (top to bottom: shortest to longest). Thick black line indicates RTs for each trial; horizontal black line separates hits (above) and missed trials (below). **(C)** Average high gamma ERBP waveforms and single-trial high gamma waterfall plots for sites on the middle temporal gyrus (MTG) and supramarginal gyrus (SMG; **g,h**, respectively).

Figure 2B depicts simultaneous recordings from the superior temporal plane, including HG and planum temporale, of the right (non language-dominant) hemisphere of the same subject (B335). In the electrode that was implanted to target the insula based on clinical requirements (contact 162; see inset in Figure 2B), three contacts (163, 164 and 165) came to reside within HG and three other (166, 167 and 168) in planum temporale. Similar to the simultaneous recordings from the left superior temporal plane (see Figure 2A), responses exhibited no task- or target-related effect and, once again, the most lateral electrodes showed slower and less robust responses. Nearly identical response profiles were obtained from the electrodes positioned along the axis of the right HG (contacts 137–144). Once again, there was no systematic effect of task or target on the responses preceding button press. Responses time-locked to the button press were observed on several sites within posteromedial HG (contacts 137 and 139) and represent increases in high gamma ERBP likely elicited by the faint sound associated with the button press. Activity at the most lateral site within HG appeared to show modest target-related effects that preceded the button press (site 144). Such task, but not target, effects were also noted within anterolateral HG in one other subject (R334). Response patterns were similar to those simultaneously recorded from non-primary auditory cortex on the lateral STG in the same subject (see e.g., site e in Figure 3A).

The exemplary data shown in Figure 2 were consistent across all but one subject (R320). In that subject, responses to the words were largest during the first recording block (complex tone detection task). This profile likely represents an order effect, wherein responses to the same stimuli can become attenuated over the course of multiple experimental blocks, as has previously been reported in a different target detection paradigm (Nourski et al., 2015).

### STG

In contrast to posteromedial HG, prominent task-related effects were observed on posterior and middle portions of the lateral STG. This is illustrated in Figure 3A, which depicts high gamma ERBP envelopes at all sites overlying the right STG in subject R334. At multiple recording sites on STG, high gamma responses elicited by words were larger during semantic classification tasks than during the tone detection task (e.g., sites d,e). Thus, high gamma activity increased above and beyond that seen in the tone task when acoustic processing was sufficient, likely reflecting additional phonemic processing necessary for completion of the semantic tasks (e.g., Mesgarani et al., 2014).

At sites reflecting a task effect (Figure 3, sites d,e), no systematic difference was observed between responses to target and non-target words prior to the behavioral response (red and green plots, respectively; the distribution of behavioral response times is indicated by box-and-whiskers plots). At all other sites on the STG, no systematic effects of task or target were observed (e.g., sites a,f), paralleling what was seen in posteromedial HG. The nearly identical high gamma envelopes obtained in the three recording blocks (blue, green, red plots) emphasize the stability and reliability of the neural activity.

Multiple sites featured target-specific increases in high gamma power that followed the behavioral response (e.g., sites c–e; Figure 3B). While these responses may have been elicited by the low-intensity click associated with the button press, it would be unusual that this later activity, if driven exclusively by the click sound, would be stronger in magnitude than that associated with the target stimulus (as seen in site d). While this response might reflect feedback from other brain regions such as PFC, future studies using a silent behavioral-response reporting device will be required to clarify this issue.

Early increases in high gamma activity occurred whether or not the subject detected target stimuli. The horizontal black lines in waterfall plots in Figure 3B demarcate the trials associated with behavioral responses (sorted by RT), from trials where the subject failed to behaviorally respond to the targets. The magnitude of short latency increases in high gamma activity preceding behavioral responses did not reliably differentiate whether or not the subject responded to the target words (e.g., sites a,e). In contrast, missed trials and those that were followed by slower behavioral responses were associated with suppression of high gamma that followed the initial excitatory response and preceded the behavioral response when present. Additional work will be required to determine whether this suppression plays a role in determining target detection or the RT of behavioral responses.

### MTG and SMG

**Figure 4:**
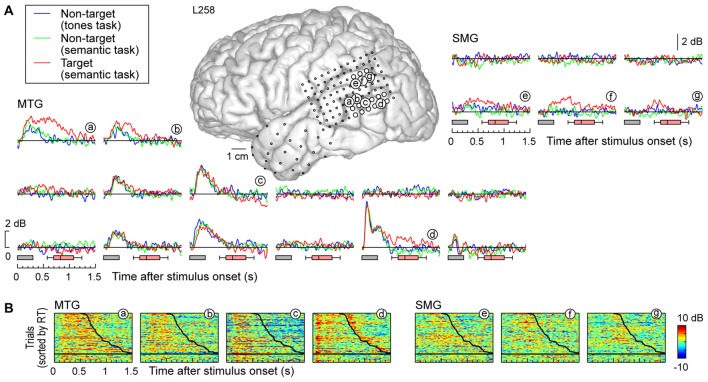
**High gamma responses recorded from left MTG and SMG in subject L258. (A)** Average high gamma ERBP waveforms. **(B)** Single-trial high gamma waterfall plots. See legend of Figures 3A,B for details.

Response patterns in MTG and SMG were markedly different from those on the STG. These differences are illustrated in Figure 3C, which depicts exemplary responses from the MTG (site g) and SMG (site h), recorded with the same electrode array that provided STG coverage in this subject (see Figure 3A). On MTG, responses were target-specific, preceded the behavioral response by several hundred milliseconds, and dissipated after the behavioral response. At site g, no response was observed when the subject failed to respond to the target. Sites over the MTG could also show early responses (albeit with a longer onset latency compared to the STG) that were neither task nor target-specific, and be followed by later target-specific activity preceding the behavioral response (Figure 4, sites a,b,d). This later activity was variable with regard to its persistence or dissipation following the button press (Figure 4B).

Increases in high gamma activity on the SMG shared similar properties with those recorded from the MTG. Specifically, early activity, whose onset was later than that on the STG, could be seen in response to the stimuli regardless of the task or target status (e.g., Figure 3C, site h). Later activity was target-specific, also preceded the behavioral response, and was absent on missed target trials (waterfall plots in Figure 3C). As for the MTG (e.g., Figure 3C, site g), non-specific responses on the SMG could be absent despite the presence of target-specific and later responses that preceded the behavioral response (Figure 4, sites e, g and g).

### PFC

**Figure 5:**
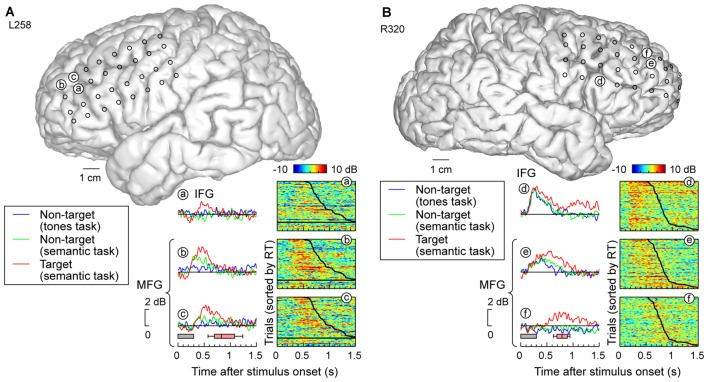
**High gamma responses recorded from prefrontal cortex (PFC; inferior and middle frontal gyri; IFG and MFG). (A)** Data from left (language-dominant) hemisphere (subject L258). **(B)** Data from right (language non-dominant) hemisphere (subject R320). See legend of Figures 3A,B for details.

Grid coverage often extended beyond the temporal lobe to include recording sites overlying ventro- and dorsolateral PFC on IFG and MFG, respectively. Response patterns were qualitatively similar to those recorded on MTG and SMG (Figure 5). Specifically, recording sites on both IFG and MFG could exhibit early responses that were not modulated by task or target status, yet be followed by increased activity specific for target stimuli (e.g., site d, Figure 5B). At the other extreme, activity could be confined to the targets (e.g., sites a,f in Figure 5). Many sites demonstrated an intermediate or graded pattern wherein responses were the largest to the target words, smallest in the tone detection task (where word categorization was not explicitly required), and intermediate for non-target words in the semantic task, where word categorization was required (e.g., sites b,c,e in Figure 5).

### Spatiotemporal Distribution of Task and Target Effects

**Figure 6:**
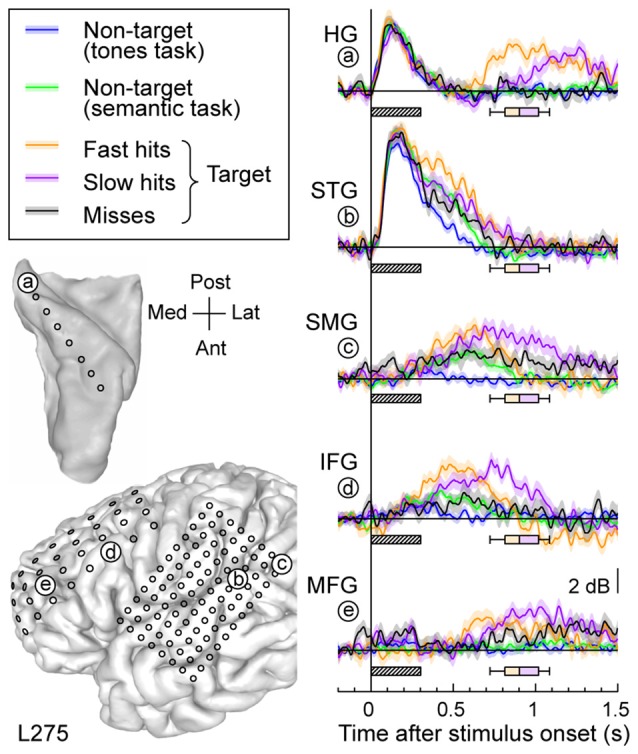
**Relative timing of high gamma responses across task and target conditions and brain regions.** Representative data from subject L275. Left column, top: MRI reconstruction of the top-down view of left superior temporal plane depicting the location of the electrode trajectory and representative recording site in posteromedial HG (site “**a**”). Left column, bottom: MRI reconstruction of the lateral view of the left cerebral hemisphere depicting the electrode coverage and location of the representative recording sites in STG, SMG, IFG and MFG (sites “**b**” through “**e**”, respectively). Right column: average high gamma waveforms recorded for the five exemplary sites (“**a**” through “**e**”). Responses were averaged across trials when words were non-targets in a tone detection task (blue), non-targets in a semantic categorization task (green) and targets in a semantic categorization tasks. For the latter condition, responses were plotted separately for trials that were associated with fast behavioral responses (orange), slow responses (purple) and misses (black). Lines and shaded areas represent mean high gamma ERBP and its standard error. Shaded boxes underneath high gamma waveforms represent stimulus duration (300 ms); box-and-whiskers plots denote the timing of button presses to the target stimuli (median, 10th, 25th, 75th and 90th percentile).

Response patterns in auditory, auditory-related and prefrontal areas could be modulated by the subject’s behavioral performance (Figure 6). High gamma activity to the target words was segregated into bins according to whether the response time was shorter (fast hits) or longer (slow hits) than the across-trial median value, or whether the subject failed to respond to the target altogether (misses). Short latency responses in auditory cortex were not strongly modulated by the subject’s performance (Figure 6, sites a,b). Longer latency high gamma activity was modulated in parallel with behavioral performance. Fast hits were associated with earlier response increases compared to slow hits, and both behavioral performance types were associated with larger responses than when the subject missed the target. The onset of this performance-related activity was similar in timing across the lateral STG, SMG and IFG. It preceded increased activity in posteromedial HG (site a) and MFG (site f).

**Figure 7:**
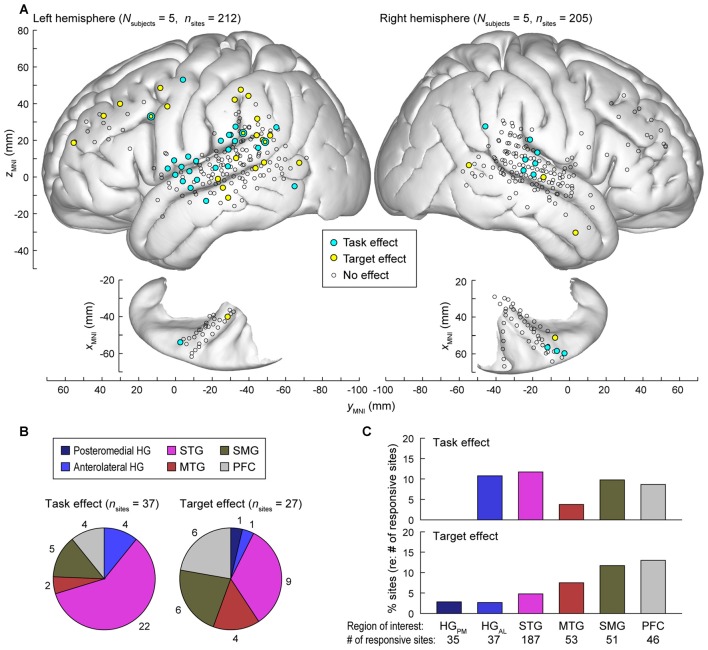
**Summary of task and target effects across the nine subjects. (A)** Spatial distribution of task and target effects (blue and yellow symbols, respectively). Data from all subjects (212 sites in the left hemisphere and 205 sites in the right hemisphere; left and right panels, respectively) are plotted in Montreal Neurological Institute (MNI) coordinate space and projected onto FreeSurfer average template brain. Open symbols indicate sites within the regions of interest that exhibited significant high gamma responses to the word stimuli (see “Materials and Methods” Section), but did not exhibit either task or target effect at *p* = 0.05 significance level (false discovery rate (FDR)-corrected). **(B)** Distribution and number of sites exhibiting significant task and target effects (left and right pie chart, respectively) across regions of interest. **(C)** Percentage of responsive sites that exhibited significant task and target effects (top and bottom graph, respectively) within each region of interest. HG_PM_, posteromedial HG; HG_AL_, anterolateral HG.

Data from all nine subjects (ten hemispheres; five left) were plotted in standard MNI coordinates in order to characterize the spatial distribution of task and target effects. Out of 784 electrode sites in HG, STG, MTG, SMG and PFC across the nine subjects, 417 (53.2%) exhibited significant responses to words in at least one of the three tested experimental conditions i.e., (non-target in tone detection or semantic task, or target; see “Materials and Methods” Section). Locations of these electrode sites are shown in Figure 7A, projected onto the template FreeSurfer brain (sites denoted by circles of all sizes and colors).

Significant hemispheric differences were noted for both task and target effects. Task effects, evaluated at *p* = 0.05 significance level (FDR-corrected), were more prominent in the left hemisphere (27/212 responsive sites) than in the right (10/205 sites; Chi-square test *p* = 0.008), indicating that a task explicitly requiring speech decoding engages the language-dominant hemisphere more than a tone target detection task. Target effects were also more prominent in the left hemisphere (23/212 sites) than in the right (4/205 sites; Chi-square test *p* < 0.001). Subject L292, who had left hemisphere electrode coverage, had right hemispheric language dominance as determined by the Wada test. None of the 14 responsive sites (5 in HG, 6 on STG, 1 on MTG, 1 on SMG and 1 on IFG) located over the left (non-dominant) cortex in this subject exhibited a significant task or target effect, further emphasizing the hemispheric asymmetry for task and target effects.

The majority of recording sites where activity was modulated by the task (22 out of 37) were located on the STG (Figure 7B, left panel). In contrast, target-related effects were more common outside of auditory cortex (MTG, SMG and PFC, Figures 7B,C). None of the seven responsive sites within the right planum temporale in subjects R334 and B335 exhibited either effect.

**Figure 8:**
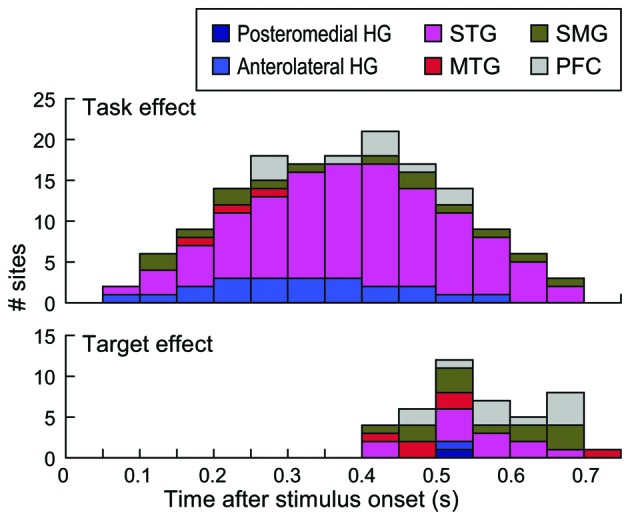
**Timing of task and target effects.** Stacked bars denote the number of sites within each of the six regions of interest across all subjects that exhibited a significant task or target effect (top and bottom graph, respectively) within time intervals from 50–100 to 700–750 ms after stimulus onset. Intervals up to 550 ms included data from all nine subjects, because this window preceded behavioral responses in all subjects. Time windows beyond 550 ms reflect data progressively fewer subjects based on their RTs (see “Materials and Methods” Section).

Timing of task and target effects is depicted in Figure 8. Time windows that overlapped with behavioral responses in each subject were not included in this analysis (see “Materials and Methods” Section). This was done to ensure that the effects were not confounded by activity elicited by the motor response or auditory activity possibly elicited by the sound of the button press. Task effects were maximal between 400 ms and 450 ms after stimulus onset, with the majority of the sites that exhibited this effect being located on the STG (see also Figure 7A). Target effects occurred later than the task effect and were more common outside the STG.

## Discussion

The current study used intracranial recordings to examine auditory selective attention when subjects listened to a single sound stream and behaviorally responded to either an acoustic or semantic target. This study expands upon previous intracranial work where stimuli were two consonant-vowel syllables, a semivowel and two vowels presented in a single-stream target detection paradigm (Chang et al., 2011). A modest preference for target syllables was found in high gamma activity recorded from the STG, whereas activity overlying PFC was selective for targets. In the present study, we took this line of research further by: (1) introducing a semantic classification task and contrasting it with a non-speech target detection task; (2) examining activity in auditory-related cortex on MTG and SMG; and (3) comparing activity between language-dominant and non-dominant hemispheres. Words, presented in the context of a semantic category task, should engage broader brain networks than those involved in strictly acoustic or phonemic processing (Corina et al., 2010). We found significant modulation of activity in auditory, auditory-related cortex, and PFC based on task and target conditions.

By using unique exemplars for each word, having different target types (animal and number) and including novel target stimuli in each task block, we aimed to minimize the subjects’ reliance exclusively on lower level acoustic or phonemic cues while performing semantic categorization tasks. However, it must be acknowledged that subjects could have used acoustic or phonemic cues when performing the semantic tasks, as the limited word set would have promoted the relevance of these cues. This possibility does not detract from the current findings, but does mandate that caution be exercised in data interpretation.

The paradigm used in the present study implicitly required Go/NoGo decisions to be made. Multiple electro- and magnetoencephalography studies have examined neural activity associated with Go/NoGo paradigms (e.g., Simson et al., 1977; Smith and Douglas, 2011; Nakata et al., 2013). In our study, Go/NoGo effects would be expected to occur in all task conditions, including tone detection and semantic categorization. Thus, increases in neural activity reported here related to semantic categorization tasks should reflect processes beyond more basic Go/NoGo effects.

### Theoretical Implications of Auditory Selective Attention

Current results bear directly upon theoretical models of auditory selective attention. Specifically, our findings generally support the “gain” theory of auditory selective attention at the level of the auditory cortex (Hillyard et al., 1973). The “gain” theory posits that attended stimuli or stimulus attributes elicit larger responses whereas unattended stimuli or their attributes are associated with a reduction or inhibitory gating of neural activity at an early stage of sensory processing (Giard et al., 2000). Empirical investigations supporting the “gain” theory of auditory selective attention have generally been performed using dichotic listening paradigms wherein the listener attends to one stream and ignores the other (e.g., Hillyard et al., 1973; Mesgarani and Chang, 2012). The current paradigm contained only one stream, with the degree of attention being modulated by tasks requiring lesser (tone targets) or greater (semantic targets) amount of speech processing. At the level of auditory cortex, increases in high gamma activity that paralleled task demands were seen relatively early after stimulus onset, supporting the “gain” theory of auditory selective attention. This task effect was observed on the anterolateral HG and STG, where numerous sites showed significantly increased responses to words target was a semantic category compared to when tones were targets (see Figure 7).

On the other hand, the “attentional trace” model of auditory selective attention can also account for increased neural activity associated with semantic categorization tasks (Näätänen, 1982, 1990). The model predicts that stimuli that are more closely related to the target (e.g., words in a semantic categorization task) will require a longer comparison process than stimuli that are highly dissimilar (e.g., tones in a semantic categorization task; Giard et al., 2000). It would thus be predicted that responses to words would be graded based upon both the task demands and the target status. This prediction is supported by activity at specific sites within PFC (e.g., b,e in Figure 5). When tones were targets, short latency responses to words were brief in duration, indicating their rapid rejection as a non-target. More prolonged responses occurred to non-target words in semantic categorization tasks, suggesting that a longer comparison process was required to identify whether the word belonged to the target category. Overall, our data support both theories in a region-specific manner, with auditory and prefrontal areas primarily operating in a way consistent with predictions of the “gain” theory and “attentional trace” model, respectively.

### Posteromedial HG

Anatomical and physiological studies have concluded that posteromedial HG is part of human core auditory cortex, likely representing a portion of A1 (e.g., Galaburda and Sanides, 1980; Liegeois-Chauvel et al., 1991; Talavage et al., 2000; Hackett et al., 2001; Morosan et al., 2001). In the present study, high gamma activity within posteromedial HG was not modulated by task, and target effects were late, paralleling the timing of the behavioral response. The absence of evidence for task modulation at this level of cortical processing differs from previous human functional neuroimaging reports (Woods et al., 2011; Da Costa et al., 2013; Riecke et al., 2016). However, attention-related modulations within posteromedial HG was not observed in a meta-analysis of auditory attention fMRI studies published prior to 2013 (Alho et al., 2014). One reason for this discrepancy may be the choice of selective attention tasks used to examine core auditory cortex. In the current study, comparisons were made between acoustic and semantic tasks. Both require acoustic processing of the stimuli regardless of whether they were the target or not. Further, both the complex tones and the speech syllables would be expected to activate comparable neural populations within the tonotopic map of A1. Overall, these considerations suggest that core auditory cortex in posteromedial HG primarily serves as a high-precision acoustic processor (Bitterman et al., 2008; Ossmy et al., 2015). Currently there is extremely limited ECoG data obtained from HG with subjects performing behavioral tasks (Steinschneider et al., 2014; Nourski et al., 2009, 2016). Parsing out the manner and degree to which core auditory cortex in humans is modulated by selective attention will ultimately require further ECoG investigations.

In contrast to current findings and previous human intracranial studies (Steinschneider et al., 2014; Nourski et al., 2015), animal studies show considerable attentional and task-related effects at the level of primary auditory cortex (e.g., Fritz et al., 2003; Brosch et al., 2005; Atiani et al., 2009; Downer et al., 2015; but see Atiani et al., 2014). Two possible explanations may account for this discrepancy. First, animal electrophysiology studies take advantage of single-neuron recordings to reveal task effects. It can be argued that these effects may be subtle enough not to be apparent at a mesoscopic level of brain function assessment offered by ECoG (Sanchez et al., 2008). Differences may also reflect increased brain resources allotted for sound processing in the human. Humans have more complex auditory cortex organization compared to other mammalian species (Hackett, 2007). The increased expanse of the auditory cortex may allow attentional modulation to primarily occur across higher areas of the auditory cortex, allowing lower auditory cortical areas (i.e., core) to maintain a more precise representation of acoustic stimulus attributes regardless of task (e.g., Bitterman et al., 2008; Ossmy et al., 2015).

### Anterolateral HG

Three characteristics of activity in our limited sample of anterolateral HG are noteworthy. First, responses in anterolateral HG were of lower amplitude when compared to activity in both posteromedial HG and on STG, paralleling previous studies that utilized passive-listening (e.g., Brugge et al., 2008, 2009) and active-listening (e.g., Nourski et al., 2009) paradigms. Second, response onset latencies in this cortical region were typically longer than those occurring in the more posteromedial portion of HG, again paralleling previous studies (Nourski et al., 2014). Third, despite the lower magnitude and longer latency, target modulation preceded the behavioral response and was stronger and earlier than that occurring in the posteromedial HG (see also Steinschneider et al., 2014). It is of note that much stronger activation of anterolateral HG, comparable in magnitude to that seen in posteromedial HG, has been observed during conversation-based paradigms (Nourski et al., 2016). This may reflect in part attentional load placed on the subject by verbal communication with the interviewer. Obviously, much work is required to further characterize this brain area and its contribution to auditory selective attention.

### STG

The key finding related to neural activity on STG is the transformation of response patterns relative to activity in posteromedial HG. Prominent task effects emerge at this level, wherein high gamma activity elicited by words can be larger when they are relevant to task requirements. When the task was to identify tone targets, it was not necessary to process the phonemic content nor the meaning of the non-target words, and neural responses were accordingly smaller in magnitude. Increased high gamma responses to words during semantic classification tasks began soon after onset of the stimulus, and peaked prior to the earliest behavioral response. This increased activity may reflect acoustic-phonemic transformations necessary to perform the word-related tasks (Vigneau et al., 2006; Chang et al., 2010; Corina et al., 2010; Turkeltaub and Coslett, 2010; Alho et al., 2014). Target effects, when present in the current study, were less commonly seen than task effects and often occurred after the behavioral response.

### MTG and SMG

Further transformations in neural activity occurred in MTG, where target effects were more prominent than task effects. Target effects began later than activity occurring on the STG yet preceded the behavioral response. These results demonstrate that this target-related activity on MTG either reflects processing necessary to perform the semantic classification task or is driven by inputs from regions that do. Current neuroanatomical models of speech processing support the importance of the language-dominant MTG for lexical access and semantic classification of words (reviewed in Lau et al., 2008). The presence of target effects in the MTG supports this model. A prime example of these roles is provided by intraoperative electrical stimulation of the language-dominant MTG, where semantic paraphasias occur without phonological errors (Corina et al., 2010). Further, the left-lateralized MTG is not activated during paradigms that only require acoustic or phonetic processing, supporting the idea that the subjects in the present study primarily based their target decisions on lexico-semantic information rather than solely on acoustic or phonological cues (Hugdahl et al., 2003; Boatman, 2004; Dronkers et al., 2004; Vigneau et al., 2006; see also Hickok and Poeppel, 2007; Lau et al., 2008).

Both task and target effects were seen in the SMG. The roles of SMG in speech processing remain controversial. A meta-analysis study supports the role of SMG in phonemic perception (Turkeltaub and Coslett, 2010) and places the SMG within the posterodorsal speech processing pathway involved in audio-motor integration (Hickok and Poeppel, 2007; Rauschecker and Scott, 2009). Accordingly, electrical stimulation of both anterior and posterior portions of SMG often led to articulatory performance errors (Corina et al., 2010). However, the same study also showed that electrical stimulation of the anterior portion of SMG disrupted semantic processing, an effect consistent with target effects observed in the present study. Additionally, SMG has been suggested to play a more general role for the perception of salient stimuli (e.g., Zevin et al., 2010). Overall, data suggest that activity in SMG may subserve multiple speech-related functions as well as more general task-related demands.

### PFC

Three response patterns were identified in PFC. The first was characterized by early non-specific activation to words regardless of their task or target status, followed by target-specific activity that preceded the behavioral response and continued after the button press. The early non-specific activation indicates that PFC has access to sensory inputs regardless task or target status. The pathways by which PFC receives this information remain unknown. Multiple sites within the auditory cortex also display early activation not modulated by task or target status. Functional connectivity between lateral STG and pars triangularis of the IFG has been established using direct electrical stimulation (Garell et al., 2013), suggesting one pathway by which PFC can receive these early sensory inputs.

The second pattern was target-specific, with activity preceding the behavioral response to the target. This target-specific activity may reflect inputs from temporal auditory-related areas, including MTG and SMG, as significant target effects generally occurred earlier in these regions (see Figure 8). Target effects that occur earlier in the temporal lobe suggest serial processing mechanisms for these attentive and non-automatic activations from the auditory cortex. In the MTG, this processing is envisioned to occur along the anteroventral auditory cortical processing pathway, whereas target-specific effects within SMG would represent activation along the posterodorsal pathway to PFC (Hickok and Poeppel, 2007; Rauschecker and Scott, 2009).

The third pattern showed a more graded effect, wherein non-target words elicited larger responses during a semantic classification task than when the task was tone detection, and target words elicited larger responses than non-targets in either task. Thus, neural activity in PFC differentiates between targets, relevant non-targets and non-relevant non-targets. This graded pattern of neural activity most closely relates to predictions made by the “attentional trace” model of selective auditory attention, with non-target stimuli being progressively discarded as potential targets based upon the degree of their dissimilarity with target sounds.

It is not possible at this time to draw firm conclusions regarding the differential roles of IFG and MFG and their subdivisions in speech processing within the context of auditory selective attention (e.g., Vigneau et al., 2006; Sahin et al., 2009). Our subject sample had a relatively sparse coverage of PFC. Further, high gamma activity in these regions, when present, tended to be of lower magnitude than that recorded from auditory cortical areas (see Figure 6). Finally, recordings were made from the hemisphere ipsilateral to the hand making the button press. Activity in the frontal lobe must at some point become greater in the hemisphere that controls the motor response (button press). Unfortunately, the one subject in the present study with bilateral electrode placement (B335) lacked coverage of the PFC. Future studies will address this issue by including both ipsi- and contralateral hand motor response conditions.

### Relationship Between High Gamma Activity and Task Performance

Each studied brain region had specific patterns of high gamma activity in relation to task performance. Responses in posteromedial HG that occurred prior to behavioral response were not predictive of the subjects’ task performance. This observation parallels similar findings observed using a tone detection task (Nourski et al., 2015) and a sentence comprehension task (Nourski et al., 2009). Within STG, fast hits could be associated with increased high gamma activity compared to slow hits or misses (see Figure 6, site b). Different patterns emerged in MTG, where activity could be of shorter latency for fast hits relative to slow hits, while misses were not associated with a response at all (see Figure 3C). Alternatively, duration of high gamma responses preceding the response on MTG could vary with RT, with shorter-duration activity being associated with faster behavioral responses (e.g., Figure 4, sites a,b). Both patterns seen in the MTG were also observed in the SMG and PFC. Differential profiles across the cortical regions are reminiscent of those seen in the monkey, with activity in the STG (field AL in the monkey) being more associated with the categorization of the sounds as target/non-target, whereas auditory-related cortex and PFC being more associated with behavioral decisions (Tsunada et al., 2011).

### Hemispheric Asymmetry

There was a significant left hemisphere bias in the manifestation of both task and target effects (see Figure 7A). Tasks involving semantic categorization more prominently engaged the language-dominant hemisphere than a tone target detection task. This observation parallels the extensive literature on hemispheric asymmetries that occur during speech processing (e.g., Vigneau et al., 2006, 2011; Rauschecker and Scott, 2009). The asymmetry, however, should not be interpreted as indicating that the non-dominant hemisphere is not involved in at least phonemic processing (e.g., Hickok and Poeppel, 2007), and current data showing statistically significant increases in task-related activity in the right STG support this interpretation. It should be noted that shifts in hemispheric activation at the level of STG are not restricted to semantic and lexical processing, but can occur based on acoustic stimulus attributes (Brechmann and Scheich, 2005; von Kriegstein et al., 2010). Functional neuroimaging has shown that the non-dominant MTG is activated when lexical access is required (Hugdahl et al., 2003). Despite adequate coverage of the non-dominant MTG in the current study, we failed to identify significant target effects that would indicate lexical and semantic processing. This reason for this discrepancy is unclear, but may represent differences related to experiment design or methodology used to assess neural activity.

### Future Directions

The current study illustrates the utility of ECoG in addressing models of auditory selective attention and lays the foundation for further investigations. While the current study focused on a single-stream target detection paradigm, more extensive studies that include multi-stream target detection paradigms must be performed to further test models of auditory selective attention. Clearly, lower ECoG frequency bands need to be examined in order to clarify feedforward and feedback interactions between auditory, auditory-related and prefrontal cortical areas (van Kerkoerle et al., 2014; Jiang et al., 2015). It is encouraging that this methodology, which must by its very nature be performed in subjects with neurologic dysfunction, yields results that are congruent with findings of non-invasive neuroimaging studies carried out in healthy individuals. Integration of results obtained in human non-invasive, human intracranial and non-human invasive electrophysiology studies should provide an ever-more detailed clarification of mechanisms underlying auditory selective attention.

## Author Contributions

MS conceived the study; KVN and MS designed the study and analyzed the data; KVN and AER collected the data. KVN, MS, AER and MAH interpreted the data, wrote the manuscript, approved its final version and agreed to be accountable for all aspects of the work.

## Funding

This study was supported by National Institutes of Health (NIH) grants R01-DC04290, UL1RR024979, National Science Foundation (NSF) grant CRCNS-IIS-1515678, and the Hoover Fund.

## Conflict of Interest Statement

The authors declare that the research was conducted in the absence of any commercial or financial relationships that could be construed as a potential conflict of interest.

## Footnotes

^1^http://librivox.org/

^2^http://www.neurobs.com/

## Abbreviations

AEP: averaged evoked potential
ECoG: Electrocorticography
fMRI: functional magnetic resonance imaging
HG: Heschl’s gyrus
IFG: inferior frontal gyrus
MFG: middle frontal gyrus
MNI: Montreal Neurological Institute
MRI: magnetic resonance imaging
MTG: middle temporal gyrus
PFC: prefrontal cortex
RT: reaction time
SMG: supramarginal gyrus
STG: superior temporal gyrus.

## References

[B2] Alho K. Rinne T. Herron T. J. Woods D. L. (2014). Stimulus-dependent activations and attention-related modulations in the auditory cortex: a meta-analysis of fMRI studies. Hear. Res. 307, 29–41. 10.1016/j.heares.2013.08.001 23938208

[B4] Atiani S. David S. V. Elgueda D. Locastro M. Radtke-Schuller S. Shamma S. A.. (2014). Emergent selectivity for task-relevant stimuli in higher-order auditory cortex. Neuron 82, 486–499. 10.1016/j.neuron.2014.02.029 24742467 PMC4048815

[B5] Atiani S. Elhilali M. David S. V. Fritz J. B. Shamma S. A. (2009). Task difficulty and performance induce diverse adaptive patterns in gain and shape of primary auditory cortical receptive fields. Neuron 61, 467–480. 10.1016/j.neuron.2008.12.027 19217382 PMC3882691

[B6] Benjamini Y. Hochberg Y. (1995). Controlling the false discovery rate: a practical and powerful approach to multiple testing. J. R. Stat. Soc. B 57, 289–300.

[B7] Bitterman Y. Mukamel R. Malach R. Fried I. Nelken I. (2008). Ultra-fine frequency tuning revealed in single neurons of human auditory cortex. Nature 451, 197–201. 10.1038/nature06476 18185589 PMC2676858

[B8] Boatman D. (2004). Cortical bases of speech perception: evidence from functional lesion studies. Cognition 92, 47–65. 10.1016/j.cognition.2003.09.010 15037126

[B9] Bregman A. S. (1990). Auditory Scene Analysis: The Perceptual Organization of Sound. Cambridge MA: MIT Press.

[B10] Brosch M. Selezneva E. Scheich H. (2005). Nonauditory events of a behavioral procedure activate auditory cortex of highly trained monkeys. J. Neurosci. 25, 6797–6806. 10.1523/JNEUROSCI.1571-05.2005 16033889 PMC6725347

[B11] Brugge J. F. Nourski K. V. Oya H. Reale R. A. Kawasaki H. Steinschneider M.. (2009). Coding of repetitive transients by auditory cortex on Heschl’s gyrus. J. Neurophysiol. 102, 2358–2374. 10.1152/jn.91346.2008 19675285 PMC2775384

[B12] Brugge J. F. Volkov I. O. Oya H. Kawasaki H. Reale R. A. Fenoy A.. (2008). Functional localization of auditory cortical fields of human: click-train stimulation. Hear. Res. 238, 12–24. 10.1016/j.heares.2007.11.012 18207680 PMC5605818

[B13] Chang E. F. Edwards E. Nagarajan S. S. Fogelson N. Dalal S. S. Canolty R. T.. (2011). Cortical spatio-temporal dynamics underlying phonological target detection in humans. J. Cogn. Neurosci. 23, 1437–1446. 10.1162/jocn.2010.21466 20465359 PMC3895406

[B14] Chang E. F. Rieger J. W. Johnson K. Berger M. S. Barbaro N. M. Knight R. T. (2010). Categorical speech representation in human superior temporal gyrus. Nat. Neurosci. 13, 1428–1432. 10.1038/nn.2641 20890293 PMC2967728

[B15] Cherry E. C. (1953). Some experiments on the recognition of speech, with one and with two ears. J. Acoust. Soc. Am. 25, 975–979. 10.1121/1.1907229

[B16] Cogan G. B. Thesen T. Carlson C. Doyle W. Devinsky O. Pesaran B. (2014). Sensory-motor transformations for speech occur bilaterally. Nature 507, 94–98. 10.1038/nature12935 24429520 PMC4000028

[B17] Corina D. P. Loudermilk B. C. Detwiler L. Martin R. F. Brinkley J. F. Ojemann G. (2010). Analysis of naming errors during cortical stimulation mapping: implications for models of language representation. Brain Lang. 115, 101–112. 10.1016/j.bandl.2010.04.001 20452661 PMC3247200

[B18] Crone N. E. Boatman D. Gordon B. Hao L. (2001). Induced electrocorticographic gamma activity during auditory perception. Clin. Neurophysiol. 112, 565–582. 10.1016/s1388-2457(00)00545-9 11275528

[B19] Crone N. E. Sinai A. Korzeniewska A. (2006). High-frequency gamma oscillations and human brain mapping with electrocorticography. Prog. Brain Res. 159, 275–295. 10.1016/s0079-6123(06)59019-3 17071238

[B20] Da Costa S. van der Zwaag W. Miller L. M. Clarke S. Saenz M. (2013). Tuning in to sound: frequency-selective attentional filter in human primary auditory cortex. J. Neurosci. 33, 1858–1863. 10.1523/JNEUROSCI.4405-12.2013 23365225 PMC4340971

[B21] Dijkstra K. Brunner P. Gunduz A. Coon W. Ritaccio A. L. Farquhar J.. (2015). Identifying the attended speaker using electrocorticographic (ECoG) signals. Brain Comput. Interfaces (Abingdon) 2, 161–173. 10.1080/2326263X.2015.1063363 26949710 PMC4776341

[B22] Ding N. Simon J. Z. (2012). Emergence of neural encoding of auditory objects while listening to competing speakers. Proc. Natl. Acad. Sci. U S A 109, 11854–11859. 10.1073/pnas.1205381109 22753470 PMC3406818

[B23] Downer J. D. Niwa M. Sutter M. L. (2015). Task engagement selectively modulates neural correlations in primary auditory cortex. J. Neurosci. 35, 7565–7574. 10.1523/JNEUROSCI.4094-14.2015 25972181 PMC4429156

[B24] Dronkers N. F. Wilkins D. P. Van Valin R. D. Jr. Redfern B. B. Jaeger J. J. (2004). Lesion analysis of the brain areas involved in language comprehension. Cognition 92, 145–177. 10.1016/j.cognition.2003.11.002 15037129

[B25] Edwards E. Soltani M. Kim W. Dalal S. S. Nagarajan S. S. Berger M. S.. (2009). Comparison of time-frequency responses and the event-related potential to auditory speech stimuli in human cortex. J. Neurophysiol. 102, 377–386. 10.1152/jn.90954.2008 19439673 PMC2712274

[B26] Egeth H. (1967). Selective attention. Psychol. Bull. 67, 41–57. 10.1037/h0024088 5340786

[B27] Fritz J. Shamma S. Elhilali M. Klein D. (2003). Rapid task-related plasticity of spectrotemporal receptive fields in primary auditory cortex. Nat. Neurosci. 6, 1216–1223. 10.1038/nn1141 14583754

[B28] Galaburda A. Sanides F. (1980). Cytoarchitectonic organization of the human auditory cortex. J. Comp. Neurol. 190, 597–610. 10.1002/cne.901900312 6771305

[B402] Garell P. C. Bakken H. Greenlee J. D. W. Volkov I. Reale R. A. Oya H. (2013). Functional connection between posterior superior temporal gyrus and ventrolateral prefrontal cortex in human. Cereb. Cortex. 23, 2309–2321. 10.1093/cercor/bhs220 22879355 PMC3767955

[B29] Garofolo J. S. Lamel L. F. Fisher W. M. Fiscus J. G. Pallett D. S. Dahlgren N. L. (1993). TIMIT Acoustic-Phonetic Continuous Speech Corpus. Philadelphia: Linguistic Data Consortium.

[B30] Giard M. H. Fort A. Mouchetant-Rostaing Y. Pernier J. (2000). Neurophysiological mechanisms of auditory selective attention in humans. Front. Biosci. 5, D84–D94. 10.2741/giard 10702372

[B31] Gomes H. Duff M. Ramos M. Molholm S. Foxe J. J. Halperin J. (2012). Auditory selective attention and processing in children with attention-deficit/hyperactivity disorder. Clin. Neurophysiol. 123, 293–302. 10.1016/j.clinph.2011.07.030 21839675 PMC3232335

[B32] Greimel E. Trinkl M. Bartling J. Bakos S. Grossheinrich N. Schulte-Körne G. (2015). Auditory selective attention in adolescents with major depression: an event-related potential study. J. Affect. Disord. 172, 445–452. 10.1016/j.jad.2014.10.022 25451449

[B33] Hackett T. A. (2007). “Organization and correspondence of the auditory cortex of humans and nonhuman primates,” in Evolution of Nervous Systems (Vol. 4: Primates), ed. Kaas J. H. (New York, NY: Academic Press), 109–119.

[B34] Hackett T. A. Preuss T. M. Kaas J. H. (2001). Architectonic identification of the core region in auditory cortex of macaques, chimpanzees, and humans. J. Comp. Neurol. 441, 197–222. 10.1002/cne.1407 11745645

[B36] Hickok G. (2009). The functional neuroanatomy of language. Phys. Life Rev. 6, 121–143. 10.1016/j.plrev.2009.06.001 20161054 PMC2747108

[B35] Hickok G. Poeppel D. (2007). The cortical organization of speech processing. Nat. Rev. Neurosci. 8, 393–402. 10.1038/nrn2113 17431404

[B37] Hillyard S. A. Hink R. F. Schwent V. L. Picton T. W. (1973). Electrical signs of selective attention in the human brain. Science 182, 177–180. 10.1126/science.182.4108.177 4730062

[B38] Howard M. A. III Volkov I. O. Granner M. A. Damasio H. M. Ollendieck M. C. Bakken H. E. (1996). A hybrid clinical-research depth electrode for acute and chronic *in vivo* microelectrode recording of human brain neurons. Technical note. J. Neurosurg. 84, 129–132. 10.3171/jns.1996.84.1.0129 8613821

[B39] Howard M. A. Volkov I. O. Mirsky R. Garell P. C. Noh M. D. Granner M.. (2000). Auditory cortex on the human posterior superior temporal gyrus. J. Comp. Neurol. 416, 79–92. 10.1002/(SICI)1096-9861(20000103)416:1<79::AID-CNE6>3.0.CO;2-2 10578103

[B40] Hugdahl K. Thomsen T. Ersland L. Rimol L. M. Niemi J. (2003). The effects of attention on speech perception: an fMRI study. Brain Lang. 81, 37–48. 10.1016/s0093-934x(02)00500-x 12681347

[B41] Jiang H. Bahramisharif A. van Gerven M. A. Jensen O. (2015). Measuring directionality between neuronal oscillations of different frequencies. Neuroimage 118, 359–367. 10.1016/j.neuroimage.2015.05.044 26025291

[B42] Kovach C. K. Gander P. E. (2016). The demodulated band transform. J. Neurosci. Methods 261, 135–154. 10.1016/j.jneumeth.2015.12.004 26711370 PMC5084918

[B84] von Kriegstein K. Smith D. R. Patterson R. D. Kiebel S. J. Griffiths T. D. (2010). How the human brain recognizes speech in the context of changing speakers. J. Neurosci. 30, 629–638. 10.1523/JNEUROSCI.2742-09.2010 20071527 PMC2824128

[B43] Lau E. F. Phillips C. Poeppel D. (2008). A cortical network for semantics: (de)constructing the N400. Nat. Rev. Neurosci. 9, 920–933. 10.1038/nrn2532 19020511

[B44] Lee A. K. Larson E. Maddox R. K. Shinn-Cunningham B. G. (2014). Using neuroimaging to understand the cortical mechanisms of auditory selective attention. Hear. Res. 307, 111–120. 10.1016/j.heares.2013.06.010 23850664 PMC3844039

[B45] Lee A. K. Rajaram S. Xia J. Bharadwaj H. Larson E. Hämäläinen M. S.. (2013). Auditory selective attention reveals preparatory activity in different cortical regions for selection based on source location and source pitch. Front. Neurosci. 6:190. 10.3389/fnins.2012.00190 23335874 PMC3538445

[B46] Liegeois-Chauvel C. Musolino A. Chauvel P. (1991). Localization of the primary auditory area in man. Brain 114, 139–151. 1900211

[B47] Mesgarani N. Chang E. F. (2012). Selective cortical representation of attended speaker in multi-talker speech perception. Nature 485, 233–236. 10.1038/nature11020 22522927 PMC3870007

[B48] Mesgarani N. Cheung C. Johnson K. Chang E. F. (2014). Phonetic feature encoding in human superior temporal gyrus. Science 343, 1006–1010. 10.1126/science.1245994 24482117 PMC4350233

[B49] Morosan P. Rademacher J. Schleicher A. Amunts K. Schormann T. Zilles K. (2001). Human primary auditory cortex: cytoarchitectonic subdivisions and mapping into a spatial reference system. Neuroimage 13, 684–701. 10.1006/nimg.2000.0715 11305897

[B50] Moses D. A. Mesgarani N. Leonard M. K. Chang E. F. (2016). Neural speech recognition: continuous phoneme decoding using spatiotemporal representations of human cortical activity. J. Neural Eng. 13:056004. 10.1088/1741-2560/13/5/056004 27484713 PMC5031534

[B400] Nakata H. Sakamoto K. Otsuka A. Yumoto M. Kakigi R. (2013). Cortical rhythm of No-go processing in humans: an MEG study. Clin. Neurophysiol. 124, 273–282. 10.1016/j.clinph.2012.06.019 22863416

[B51] Näätänen R. (1982). Processing negativity: an evoked-potential reflection of selective attention. Psychol. Bull. 92, 605–640. 10.1037/0033-2909.92.3.605 7156260

[B52] Näätänen R. (1990). The role of attention in auditory information processing as revealed by event-related potentials and other brain measures of cognitive function. Behav. Brain Sci. 13, 201–233. 10.1017/s0140525x00078407

[B53] Nir Y. Fisch L. Mukamel R. Gelbard-Sagiv H. Arieli A. Fried I.. (2007). Coupling between neuronal firing rate, gamma LFP and BOLD fMRI is related to interneuronal correlations. Curr. Biol. 17, 1275–1285. 10.1016/j.cub.2007.06.066 17686438

[B54] Noterdaeme M. Amorosa H. Mildenberger K. Sitter S. Minow F. (2001). Evaluation of attention problems in children with autism and children with a specific language disorder. Eur. Child Adolesc. Psychiatry 10, 58–66. 10.1007/s007870170048 11315537

[B401] Nourski K. V. Reale R. A. Oya H. Kawasaki H. Kovach C. K. Chen H. (2009). Temporal envelope of time-compressed speech represented in the human auditory cortex. J. Neurosci. 29, 15564–15574. 10.1523/JNEUROSCI.3065-09.2009 20007480 PMC2851231

[B59] Nourski K. V. Brugge J. F. Reale R. A. Kovach C. K. Oya H. Kawasaki H.. (2013). Coding of repetitive transients by auditory cortex on posterolateral superior temporal gyrus in humans: an intracranial electrophysiology study. J. Neurophysiol. 109, 1283–1295. 10.1152/jn.00718.2012 23236002 PMC3602837

[B55] Nourski K. V. Howard M. A. III (2015). Invasive recordings in the human auditory cortex. Handb. Clin. Neurol. 129, 225–244. 10.1016/b978-0-444-62630-1.00013-5 25726272

[B56] Nourski K. V. Steinschneider M. McMurray B. Kovach C. K. Oya H. Kawasaki H.. (2014). Functional organization of human auditory cortex: investigation of response latencies through direct recordings. Neuroimage 101, 598–609. 10.1016/j.neuroimage.2014.07.004 25019680 PMC4430832

[B57] Nourski K. V. Steinschneider M. Oya H. Kawasaki H. Howard M. A. III (2015). Modulation of response patterns in human auditory cortex during a target detection task: an intracranial electrophysiology study. Int. J. Psychophysiol. 95, 191–201. 10.1016/j.ijpsycho.2014.03.006 24681353 PMC4430839

[B58] Nourski K. V. Steinschneider M. Rhone A. E. (2016). Electrocorticographic activation within human auditory cortex during dialog-based language and cognitive testing. Front. Hum. Neurosci. 10:202. 10.3389/fnhum.2016.00202 27199720 PMC4854871

[B60] Ossmy O. Fried I. Mukamel R. (2015). Decoding speech perception from single cell activity in humans. Neuroimage 117, 151–159. 10.1016/j.neuroimage.2015.05.001 25976925

[B61] Paltoglou A. E. Sumner C. J. Hall D. A. (2009). Examining the role of frequency specificity in the enhancement and suppression of human cortical activity by auditory selective attention. Hear. Res. 257, 106–118. 10.1016/j.heares.2009.08.007 19706320

[B62] Paltoglou A. E. Sumner C. J. Hall D. A. (2011). Mapping feature-sensitivity and attentional modulation in human auditory cortex with functional magnetic resonance imaging. Eur. J. Neurosci. 33, 1733–1741. 10.1111/j.1460-9568.2011.07656.x 21447093 PMC3110306

[B64] Rauschecker J. P. Scott S. K. (2009). Maps and streams in the auditory cortex: nonhuman primates illuminate human speech processing. Nat. Neurosci. 12, 718–724. 10.1038/nn.2331 19471271 PMC2846110

[B65] Ray S. Niebur E. Hsiao S. S. Sinai A. Crone N. E. (2008). High-frequency gamma activity (80–150Hz) is increased in human cortex during selective attention. Clin. Neurophysiol. 119, 116–133. 10.1016/j.clinph.2007.09.136 18037343 PMC2444052

[B66] Reddy C. G. Dahdaleh N. S. Albert G. Chen F. Hansen D. Nourski K.. (2010). A method for placing Heschl gyrus depth electrodes. J. Neurosurg. 112, 1301–1307. 10.3171/2009.7.JNS09404 19663547 PMC3816376

[B67] Riecke L. Peters J. C. Valente G. Kemper V. G. Formisano E. Sorger B. (2016). Frequency-selective attention in auditory scenes recruits frequency representations throughout human superior temporal cortex. Cereb. Cortex 10.1093/cercor/bhw160 [Epub ahead of print]. 27230215

[B68] Sahin N. T. Pinker S. Cash S. S. Schomer D. Halgren E. (2009). Sequential processing of lexical, grammatical and phonological information within Broca’s area. Science 326, 445–449. 10.1126/science.1174481 19833971 PMC4030760

[B69] Sanchez J. C. Gunduz A. Carney P. R. Principe J. C. (2008). Extraction and localization of mesoscopic motor control signals for human ECoG neuroprosthetics. J. Neurosci. Methods 167, 63–81. 10.1016/j.jneumeth.2007.04.019 17582507

[B70] Scholes K. E. Martin-Iverson M. T. (2010). Disturbed prepulse inhibition in patients with schizophrenia is consequential to dysfunction of selective attention. Psychophysiology 47, 223–235. 10.1111/j.1469-8986.2009.00927.x 19824951

[B71] Simon J. Z. (2015). The encoding of auditory objects in auditory cortex: insights from magnetoencephalography. Int. J. Psychophysiol. 95, 184–190. 10.1016/j.ijpsycho.2014.05.005 24841996 PMC4233196

[B72] Simson R. Vaughan H. G. Jr. Ritter W. (1977). The scalp topography of potentials in auditory and visual Go/NoGo tasks. Electroencephalogr. Clin. Neurophysiol. 43, 864–875. 10.1016/0013-4694(77)90009-8 73454

[B73] Smith J. L. Douglas K. M. (2011). On the use of event-related potentials to auditory stimuli in the Go/NoGo task. Psychiatry Res. 193, 177–181. 10.1016/j.pscychresns.2011.03.002 21764566

[B74] Steinschneider M. Fishman Y. I. Arezzo J. C. (2008). Spectrotemporal analysis of evoked and induced electroencephalographic responses in primary auditory cortex (A1) of the awake monkey. Cereb. Cortex 18, 610–625. 10.1093/cercor/bhm094 17586604

[B75] Steinschneider M. Nourski K. V. Rhone A. E. Kawasaki H. Oya H. Howard M. A. III (2014). Differential activation of human core, non-core and auditory-related cortex during speech categorization tasks as revealed by intracranial recordings. Front. Neurosci. 8:240. 10.3389/fnins.2014.00240 25157216 PMC4128221

[B76] Storey J. D. (2002). A direct approach to false discovery rates. J. R. Stat. Soc. Series B Stat. Methodol. 64, 479–498. 10.1111/1467-9868.00346

[B77] Talavage T. M. Ledden P. J. Benson R. R. Rosen B. R. Melcher J. R. (2000). Frequency-dependent responses exhibited by multiple regions in human auditory cortex. Hear Res. 150, 225–244. 10.1016/s0378-5955(00)00203-3 11077206

[B78] Tsunada J. Lee J. H. Cohen Y. E. (2011). Representation of speech categories in the primate auditory cortex. J. Neurophysiol. 105, 2634–2646. 10.1152/jn.00037.2011 21346209 PMC3118748

[B79] Turkeltaub P. E. Coslett H. B. (2010). Localization of sublexical speech perception components. Brain Lang. 114, 1–15. 10.1016/j.bandl.2010.03.008 20413149 PMC2914564

[B80] van Kerkoerle T. Self M. W. Dagnino B. Gariel-Mathis M. A. Poort J. van der Togt C.. (2014). Alpha and gamma oscillations characterize feedback and feedforward processing in monkey visual cortex. Proc. Natl. Acad. Sci. U S A 111, 14332–14341. 10.1073/pnas.1402773111 25205811 PMC4210002

[B82] Vigneau M. Beaucousin V. Hervé P. Y. Duffau H. Crivello F. Houdé O.. (2006). Meta-analyzing left hemisphere language areas: phonology, semantics and sentence processing. Neuroimage 30, 1414–1432. 10.1016/j.neuroimage.2005.11.002 16413796

[B83] Vigneau M. Beaucousin V. Hervé P. Y. Jobard G. Petit L. Crivello F.. (2011). What is right-hemisphere contribution to phonological, lexico-semantic and sentence processing? Insights from a meta-analysis. Neuroimage 54, 577–593. 10.1016/j.neuroimage.2010.07.036 20656040

[B85] Whittingstall K. Logothetis N. K. (2009). Frequency-band coupling in surface EEG reflects spiking activity in monkey visual cortex. Neuron 64, 281–289. 10.1016/j.neuron.2009.08.016 19874794

[B86] Woods D. L. Herron T. J. Cate A. D. Kang X. Yund E. W. (2011). Phonological processing in human auditory cortical fields. Front. Hum. Neurosci. 5:42. 10.3389/fnhum.2011.00042 21541252 PMC3082852

[B87] Woods D. L. Herron T. J. Cate A. D. Yund E. W. Stecker G. C. Rinne T.. (2010). Functional properties of human auditory cortical fields. Front. Syst. Neurosci. 4:155. 10.3389/fnsys.2010.00155 21160558 PMC3001989

[B88] Zevin J. D. Yang J. Skipper J. I. McCandliss B. D. (2010). Domain general change detection accounts for “dishabituation” effects in temporal-parietal regions in functional magnetic resonance imaging studies of speech perception. J. Neurosci. 30, 1110–1117. 10.1523/JNEUROSCI.4599-09.2010 20089919 PMC2848500

